# Atorvastatin Attenuates Isoflurane-Induced Activation of ROS-p38MAPK/ATF2 Pathway, Neuronal Degeneration, and Cognitive Impairment of the Aged Mice

**DOI:** 10.3389/fnagi.2020.620946

**Published:** 2021-01-14

**Authors:** Pengfei Liu, Quansheng Gao, Lei Guan, Weixuan Sheng, Yanting Hu, Teng Gao, Jingwen Jiang, Yongxing Xu, Hui Qiao, Xinying Xue, Sanhong Liu, Tianzuo Li

**Affiliations:** ^1^Department of Anesthesiology, Beijing Shijitan Hospital, Capital Medical University, Beijing, China; ^2^Department of Operational Medicine, Tianjin Institute of Environmental and Operational Medicine, Tianjin, China; ^3^Department of Nephrology, Chinese PLA Strategic Support Force Characteristic Medical Center, Beijing, China; ^4^Department of Respiratory and Critical Care, Beijing Shijitan Hospital, Capital Medical University, Beijing, China; ^5^Institute of Interdisciplinary Integrative Medicine Research, Shanghai University of Traditional Chinese Medicine, Shanghai, China

**Keywords:** mitogen activated protein kinases, neuronal degeneration, reactive oxygen species, isoflurane, atorvastatin, aging

## Abstract

Isoflurane, a widely used volatile anesthetic, induces neuronal apoptosis and memory impairments in various animal models. However, the potential mechanisms and effective pharmacologic agents are still not fully understood. The p38MAPK/ATF-2 pathway has been proved to regulate neuronal cell survival and inflammation. Besides, atorvastatin, a 3-hydroxy-3-methylglutaryl coenzyme A reductase inhibitor, exerts neuroprotective effects. Thus, this study aimed to explore the influence of atorvastatin on isoflurane-induced neurodegeneration and underlying mechanisms. Aged C57BL/6 mice (20 months old) were exposed to isoflurane (1.5%) anesthesia for 6 h. Atorvastatin (5, 10, or 20 mg/kg body weight) was administered to the mice for 7 days. Atorvastatin attenuated the isoflurane-induced generation of ROS and apoptosis. Western blotting revealed a decrease in cleaved caspase-9 and caspase-3 expression in line with ROS levels. Furthermore, atorvastatin ameliorated the isoflurane-induced activation of p38MAPK/ATF-2 signaling. In a cellular study, we proved that isoflurane could induce oxidative stress and inflammation by activating the p38MAPK/ATF-2 pathway in BV-2 microglia cells. In addition, SB203580, a selected p38MAPK inhibitor, inhibited the isoflurane-induced inflammation, oxidative stress, and apoptosis. The results implied that p38MAPK/ATF-2 was a potential target for the treatment of postoperative cognitive dysfunction.

## Introduction

Inhalation anesthetics are extensively employed during surgeries in adult and aged patients. Several studies conducted in rodents have shown that exposure to inhalation anesthetics induces neurodegeneration and impairs memory and cognitive function (Zhao et al., 2010; Komita et al., 2013; Fu et al., 2020). Isoflurane is commonly used in the clinic (Kumar et al., 2019; Peyton et al., 2020; Warren et al., 2020), which has also been reported to induces neuroinflammation, neuronal apoptosis, and cognitive impairment (Cui et al., 2020; Neghab et al., 2020). Anesthesia and surgery induce systemic inflammatory response and oxidative stress, which could enter the brain through a variety of pathways [either passing directly through the blood brain barrier (BBB) or destroying the BBB] or sending signals to the brain (Otani and Shichita, 2020; Scuderi et al., 2020). The increase of proinflammatory cytokines in the brain will over-activate microglia cells and induce more inflammatory substances to be released in the brain, resulting in nerve cell damage with a vicious cycle (Bathini et al., 2020; Jeong et al., 2020). However, underlying pathways remain unclear and effective interventions are still lacking.

Both our preliminary work and previous studies found that isoflurane anesthesia could induce oxidative stress and neuroinflammation, further leading to cognitive impairment in aged mice (Neghab et al., 2020; Olufs et al., 2020). However, the potential signaling pathways involved in isoflurane-induced d neuroinflammation and apoptosis in the aged brain remain unclear. Reactive oxygen species (ROS) can activate multiple signal transduction pathways, such as c-Jun N-terminal kinases (JNKs) (Busquets et al., 2020), mitogen-activated protein kinase (p38MAPK) (Alam et al., 2019; Medeiros et al., 2020), as well as glycogen synthase kinase-3 pathway (GSK-3β) (Zhao et al., 2020), and so on, to connect intracellular reactive oxygen signaling, gene expression, and cell related functional changes (Shen et al., 2020). The p38MAPK is an important molecular signal for inflammation regulation. After the phosphorylation of p38MAPK, activating transcription factor 2 (ATF2) is phosphorylated, and the related cell surface signals are transmitted to the nucleus, leading to activation of microglia, increased transcription, and release of inflammatory factors (Zhao et al., 2016; Song et al., 2017). Thus, whether isoflurane induces neuroinflammation by activating the ROS-p38MAPK/ATF2 pathway remains to be determined.

Atorvastatin, a common lipid-lowering drug, strongly inhibits 3-hydroxy-3-methylglutaryl coenzyme A reductase and treats cognitive impairment (Kaviani et al., 2017). It protects the cognition of aged patients with neurodegenerative diseases, such as Parkinson’s and Alzheimer’s disease (Yang Y. et al., 2020). Moreover, it could inhibit amyloid-β peptide-induced cognitive impairment in rats partly by the JNK/p38MAPK pathway (Zhang et al., 2014; Shao et al., 2017), which implies that mitogen-activated protein kinase (MAPK) pathways may be associated with the neuroprotective effects of atorvastatin. However, the effect of atorvastatin on isoflurane-induced cognitive impairment in the aged brain remains unclear. Therefore, in this study, we hypothesized that atorvastatin pretreatment could inhibit isoflurane-induced apoptosis and cognitive impairment in aged mice, partly by inhibiting the ROS-p38MAPK/ATF-2 pathway.

## Materials and Methods

### Ethics Statement

The whole study was approved by the ethical committee of the Beijing Shijitan Hospital, Capital Medical University, and the procedures that we performed were in accordance with the guidelines for the use of laboratory animals issued by the National Institutes.

### Animals Groups and Treatments

Aged C57BL/6 mice (20 months old) were ordered from the Capital Medical University experimental animal center and were housed in sterile cages under standard animal housing conditions (relative humidity: 55%–60%; temperature: 22 ± 1°C; 12:12 h light/dark cycle). The mice were divided into six groups (*n* = 24 mice per group):

control group (normal saline as placebo), atorvastatin group (20 mg/kg atorvastatin treatment), isoflurane group (1.5% isoflurane exposure for 6 h), 5 mg/kg atorvastatin + isoflurane group, 10 mg/kg atorvastatin + isoflurane group, and 20 mg/kg atorvastatin + isoflurane group.

The three doses (5, 10, or 20 mg/kg) of atorvastatin (Lipitor Atorvastatin calcium, Pfizer) were administered orally to the mice every day for 7 days. Twenty-four hours after the last treatment, the mice received 1.5% isoflurane anesthesia for 6 h with 21% oxygen. The temperature was maintained between 33 and 35°C. The mice administered with normal saline or 21% oxygen alone were normal controls; mice that received isoflurane alone were isoflurane control; mice receiving 20 mg/kg atorvastatin alone were atorvastatin control. Then, ten mice were randomly selected from each group, to received behavioral tests 1 day after anesthesia.

### Behavior Studies

#### Open Field Tests

One day after isoflurane exposure, all mice were placed in the center of the chamber used for the open field test (50 cm × 50 cm × 40 cm), the inner walls of which were painted white. The mice were placed in there, one at a time, for 5 min, and the trajectory was observed and analyzed using a tracking software system (Shanghai Softmaze Information Technology Co., Ltd.). At the end of each test, 75% alcohol was used to eliminate the odor of the previous mouse. The whole distance of the trajectory and the time that the mice stayed in the central area were recorded and analyzed.

#### The Morris Water Maze Test

The Morris water maze (MWM) test assesses spatial training and memory. A pool with a diameter of 120 cm and a depth of 50 cm was used for the test. There are four quadrants in this pool, marked with quadrants I, II, III, and IV. A platform was placed in the third quadrant (the target quadrant) and 1 cm below the surface. The test consists of two parts: acquisition training and probe trial. Twenty-four hours after the open field test (OF), the mice underwent training for 5 consecutive days. All mice were allowed to find the platform within 60 s and stay on the platform for 10 s. The escape latency and mean speed were recorded. The mice that could not find the platform within 60 s were manually placed on the platform for 10 s. At the 6th day, the probe trial was performed. All mice were placed in the first quadrant (opposite to quadrant III) and allowed to swim for 60 s in the absence of the platform. The number of crossings of the platform, the time spent in the third quadrant, and the trajectory were recorded.

### TUNEL Fluorescent Assay

One day after isoflurane, the mice were transcardially perfused with 4% paraformaldehyde. The hippocampal tissue was isolated, embedded with paraffin, and sliced into 5 μm sections for TUNEL (terminal transferase biotinylated-dUTP nick end labeling) staining. The Dead End^TM^ fluorometric TUNEL system kit (Promega, Madison, WI, United States) was used to assess neuroapoptosis in the CA1 region of the hippocampus, according to the instructions provided (Liao et al., 2014). The apoptotic cells were imaged and calculated using NIS-Elements BR processing and analysis software (Nikon Corporation, Japan) (Liao et al., 2014). The number of apoptotic cells in the CA1 and CA3 region of the hippocampus was counted by microscopy at 400 × magnification. The results were presented as the ratio of the apoptotic cells, compared with the control group (%, of the control group).

### Cell Culture and Treatment

BV-2, a microglial cell line, (purchased from Cell Bank of Chinese Academy of Sciences, Beijing, China), was used to further illustrate the role of the p38MAPK/ATF2 pathway in isoflurane induced neuroinflammation. The cells were cultured and maintained in Dulbecco’s modified Eagle’s medium (DMEM), which contained 10% fetal bovine serum, 10 μg/mL penicillin, and 10 U/mL streptomycin, as previously reported (Wang et al., 2018; Zhu et al., 2020). The cells were cultivated under the conditions of 95% air and 5% CO_2_ at 37°C and passaged by trypsinization for further study (Wang et al., 2018).

SB203580 (Sigma, United States), a p38MAPK inhibitor, was dissolved in dimethylsulfoxide (DMSO) and DMEM before the study (Zhang et al., 2019; Liu et al., 2020). The BV-2 cells were first incubated with DMSO or 5, 10, and 20 μmol/L SB203580, diluted in DMEM. Then, the cells were exposed to 95% air and 5% CO_2_ with or without 1.5% isoflurane for 6 h in one chamber. The cells were divided into six groups for this part, including control (DMSO alone), isoflurane (1.5% isoflurane for 6 h alone), 20 μmol/L SB203580 (20 μmol/L SB203580 alone, as treatment control), 20 μmol/L SB203580 + isoflurane (20 μmol/L SB203580 + 1.5% isoflurane for 6 h), 10 μmol/L SB203580 + isoflurane (10 μmol/L SB203580 + 1.5% isoflurane for 6 h), and 5 μmol/L SB203580 + isoflurane (5 μmol/L SB203580 + 1.5% isoflurane for 6 h).

### Cell Viability and Lactate Dehydrogenase Release Assay

Cell viability was determined by the CCK-8 method (Cell Counting kit-8, Tongren Institute of Chemistry, Japan) (Zhu et al., 2020). The cells were incubated with DMEM and CCK-8 medium for 3 h, and the absorbance value was measured using a marker at a wavelength of 450 nm. Cell density was calculated using the absorbance equation. Cell survival was calculated based on the cell density and normalized by the control group (% of the control).

Lactate dehydrogenase (LDH) is abundantly present in the cytoplasm and cannot cross the cell membrane under normal conditions. When cell damage or death occurs, the LDH is released into the extracellular space. Thus, LDH activity in cell culture medium is related to cell death (Xiang et al., 2014; Zhu et al., 2020). LDH activity was detected according to the instructions of the LDH release assay kits (Promega, United States). The absorbance value was measured using a marker at a wavelength of 490 nm. Finally, the percentage of LDH release was calculated. The ratio of LDH release to the total LDH activity was considered as the percentage of cell death, as previously reported (Xiang et al., 2014).

### Immunoblotting

For the western blot analysis, the hippocampus tissue was isolated and stored at −80°C. The method was performed as described in previous studies (Liao et al., 2014; Fu et al., 2020). The hippocampi were homogenized with PMSF and RIPA lysis buffer (Beyotime, Shanghai, China) and then centrifuged at 12,000 rpm for 20 min. The BV-2 cells collected after isoflurane exposure were also lysed with RIPA lysis buffer and then centrifuged at 12000 rpm for 20 min. Both the supernatant of the tissue and cells were obtained to detect the concentration of total protein using a BCA Protein Assay Reagent Kit (Zhongshan Jinqiao Institute of Biotechnology, Beijing, China). These samples were separated by SDS-PAGE and then transferred to a polyvinylidene difluoride membrane (Invitrogen). Then, the membranes were incubated with the blocking solution and primary antibodies overnight at 4°C. The primary antibodies included: anti-Bax (1:1000, Cell Signaling Technology, United States), anti-Bcl-2 (1:1000, Cell Signaling Technology, United States), anti-caspase-3 and cleaved caspase-3 (1:1000, Cell Signaling Technology, United States), anti-p38MAPK and phospho-p38MAPK (1:1000, Cell Signaling Technology, United States), anti-ATF2 (1:1000, Abcam, United Kingdom), anti- phospho-ATF2 (1:5000, Abcam, United Kingdom), anti-nuclear factor kappa-B p56 (NF-κB p65), and phospho-NF-κB p65 (1:1000; Abcam, United Kingdom), anti-IκB-α (1:2000, Abcam, United Kingdom), and β-actin (1:2000; Abcam, United Kingdom). After being washed with TBS-Tween, the membranes were incubated with horseradish peroxidase-conjugated secondary antibodies (1:1000, ABGENT, United States). Finally, enhanced chemiluminescence (ECL, Vazyme, Nanjing, China) was used to detect the bands. The intensity of each band was quantified using ImageJ software, version 2.0.0 and normalized against β-actin.

### Measurement of ROS, MDA, and SOD

A ROS assay kit (Genmed Scientifics Inc.) was used to detect ROS levels. The kit contained a fluorogenic probe (DCFH-DA), which could react with ROS. Fluorescence was detected using a spectrofluorometer (excitation, 520 nm; emission, 490 nm). All procedures were performed according to the manufacturer’s instructions. The fluorescence intensity was observed using a microscope imaging system (Olympus America, United States).

Malondialdehyde (MDA) is a reliable indicator of lipid peroxidation while superoxide dismutase (SOD) is an endogenous scavenger of the reactive oxygen species (ROS). All procedures were performed in accordance with the manufacturer’s instructions provided along with the kits (Beyotime Biotechnology Institute, Nantong, China). The results are illustrated as the MDA concentration (nmol/mg protein) and SOD activity (U/mg).

### Enzyme-Linked Immunosorbent Assays

The levels of interleukin-1β (IL-1β) and tumor necrosis factor α (TNF-α) in the hippocampus and BV-2 cells were detected using ELISA kits (Abcam, United Kingdom). The collected hippocampus was homogenized with RIPA cracking liquid (Applygen, China) for 30 min and centrifuged at 4°C at 12000 rpm for 30 min. The supernatant was extracted for quantitative BCA protein analysis (Pierce, United States). The levels of IL-1β and TNF-α in the hippocampus (pg/mg) and BV-2 cells (pg/ml) were detected according to the manufacturer’s instructions.

### Statistical Analyses

The obtained results are reported as mean ± standard deviation (SD). SPSS 25.0 (SPSS Inc., Chicago, IL, United States) and GraphPad Prism 6.0 (La Jolla, CA) were used for statistical analysis. Two-way repeated measures analysis of variance (ANOVA) was performed to analyze the escape latency in the training period of the MWM test. Other data from the *in vitro* study (isoflurane × atorvastatin) and *in vivo* study (isoflurane × SB203580) were evaluated using two-way ANOVA followed by Bonferroni *post hoc* test or by Dunnett test if the homogeneity of variance was not met. *P*-values < 0.05 were considered to indicate statistical significance.

## Results

### Atorvastatin Prevented Cognitive Impairment Induced by Isoflurane Anesthesia in Aged Mice

**FIGURE 1:**
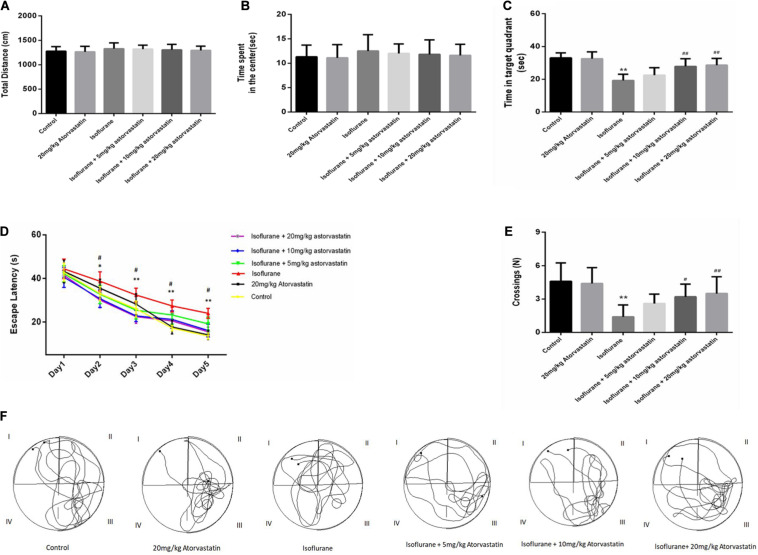
Atorvastatin prevented isoflurane induced spatial learning and memory impairment in aged mice. **(A)** The total distance of the open field. **(B)** The time spent by the mice in the center of the open field. **(C)** The time spent in the target quadrant (quadrant III) of the MWM. **(D)** The escape latency of the mice while training for the MWM test. **(E)** The number of platform crossings. **(F)** Representative trajectories of the mice in the probe trial. The values are represented as mean ± SD, (*n* = 10 per group). *P < 0.05 and **P < 0.01: Control Group versus Isoflurane Group, ^#^*P* < 0.05 and ^##^*P* < 0.01: Atorvastatin treatment (5 mg/kg, 10 mg/kg, and 20 mg/kg) + Isoflurane Group versus Isoflurane Group, ^&^*P* < 0.05 and ^&&^*P* < 0.01: Isoflurane + Atorvastatin with 5 mg/kg and 10 mg/kg Groups versus Isoflurane + 20 mg/kg Atorvastatin Group.

The open field test was used to evaluate the general behavioral performance of the mice. The results showed that isoflurane could not impair the total distance (*F* = 0.595, *P* > 0.05, Figure 1A) and the time spent in the center (*F* = 0.361, *P* > 0.05, Figure 1B), which reflect the anxiety responses. Furthermore, atorvastatin treatment with the three doses (5,10, and 20 mg.kg^–1^.d^–1^) did not change the general behavioral performance (*P* > 0.05, Figures 1A,B).

According to the MWM test, 1.5% isoflurane induced a significant increase in the escape latency during the training period (*F* = 26.569, *P* < 0.01, Figure 1D) and a decrease in the target quadrant time (*F* = 18.114, *P* < 0.01, Figure 1C) and cross-platform times (*F* = 8.301, *P* < 0.01, Figure 1E) in the probe trial compared with those of the control group. In addition, the Bonferroni *post hoc* test revealed that atorvastatin treatment (5 mg/kg) did not attenuate the isoflurane-induced decrease in the target quadrant time (*P* > 0.05, Figure 1C) and the crossing-platform times (*P* > 0.05, Figure 1E). However, both the 10 mg/kg and 20 mg/kg atorvastatin treatments ameliorated these changes in the escape latency during the training period (*P* < 0.01, Figure 1D), the target quadrant time (*P* < 0.01, Figure 1C), and cross-platform times (*P* < 0.05, Figure 1E) but not the 5 mg/kg dose.

### Atorvastatin Reduced Neuronal Apoptosis and Apoptotic Protein Activation Following Isoflurane Exposure

**FIGURE 2:**
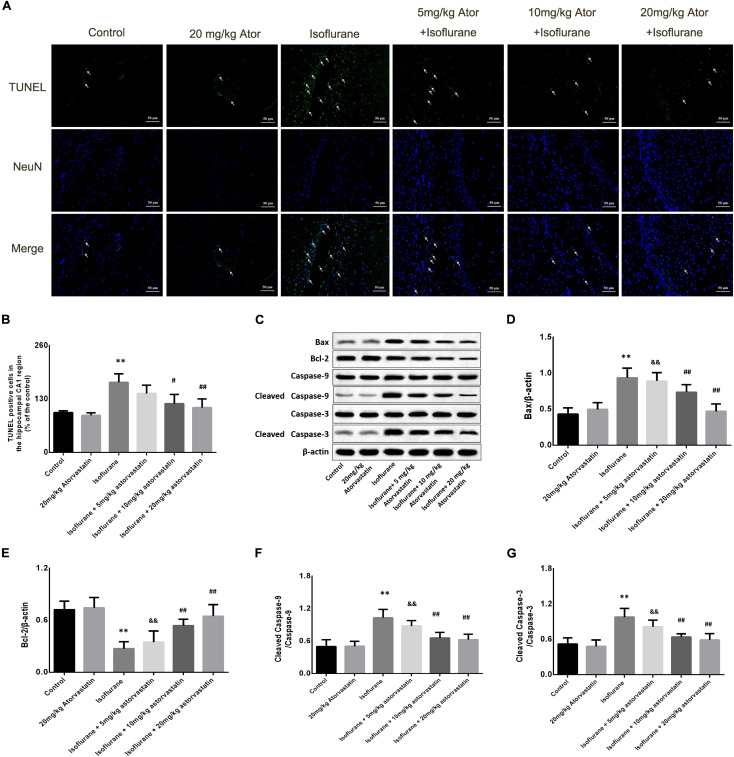
Atorvastatin reduced cellular apoptosis and related proteins in the aged mice exposed to isoflurane. **(A)** Typical images of the TUNEL staining in the hippocampal CA1 region. Scale bar = 50 mm. Cells with green color were the apoptotic cells and blue color indicated the Neu-N stained nucleus. **(B)** Statistical numbers of the apoptotic cells in the hippocampal CA1 region. **(C)** Representative western blotting of the apoptosis pathway proteins, including Bcl-2, Bcl-xl, cleaved caspase-9, and cleaved caspase-3. **(D–G)** Quantitative analysis of the levels of these proteins in the hippocampus among different groups. Atorvastatin pretreatment significantly reduced expression of Bax and enhanced expression of Bcl-2 as well as activated caspase-9 and caspase-3. The values are represented as mean ± SD, (*n* = 8 per group). *P < 0.05 and **P < 0.01: Control Group versus Isoflurane Group, ^#^*P* < 0.05 and ^##^*P* < 0.01: Atorvastatin treatment (5 mg/kg, 10 mg/kg, and 20 mg/kg) + Isoflurane Group versus Isoflurane Group, ^&^*P* < 0.05 and ^&&^*P* < 0.01: Isoflurane + Atorvastatin with 5 mg/kg and 10 mg/kg Groups versus Isoflurane + 20 mg/kg Atorvastatin Group.

In this study, TUNEL staining showed significant apoptosis in the hippocampal CA1 region of the aged mice exposed to 1.5% isoflurane (*F_*CA*1_* = 18.270, *P* < 0.01, Figures 2A,B). Atorvastatin treatment at doses of 10 and 20 mg/kg significantly attenuated isoflurane-induced apoptosis (*P* < 0.01, Figures 2A,B) but not at a dose of 5 mg/kg (*P* > 0.05, Figures 2A,B). However, there were no significant differences in the number of apoptotic cells among the three doses (*P* > 0.05, Figures 2A,B) by a Bonferroni *post hoc* test. The expression of apoptotic cascade proteins was assessed by western blotting to explore the related molecular events. Bcl-2 is an anti-apoptotic protein, while Bax is an apoptosis-related protein. In this study, isoflurane exposure significantly inhibited the expression of Bcl-2 (*F* = 26.826, *P* < 0.01, Figures 2C,D), while promoting Bax (*F* = 34.526, *P* < 0.01, Figures 2C,E) and activated caspase-9 (*F* = 27.772, *P* < 0.01, Figures 2C,F) and caspase-3 expression (*F* = 24.550, *P* < 0.01, Figures 2C,G) as demonstrated by western blotting. Pretreatment with atorvastatin (10 or 20 mg/kg) upregulated the expression of Bcl-2 and inhibited the expression of Bax, cleaved caspase-9, and cleaved caspase-3 (*P* < 0.05, Figures 2C–G). These results proved the anti-apoptotic effects of atorvastatin. In addition, atorvastatin at a dose of 5 mg/kg did not significantly affect the hippocampal levels of the four proteins (*P* > 0.05, Figures 2C–G).

### Atorvastatin Ameliorates Isoflurane-Induced Oxidative Stress and Neuroinflammation in the Hippocampus

**FIGURE 3:**
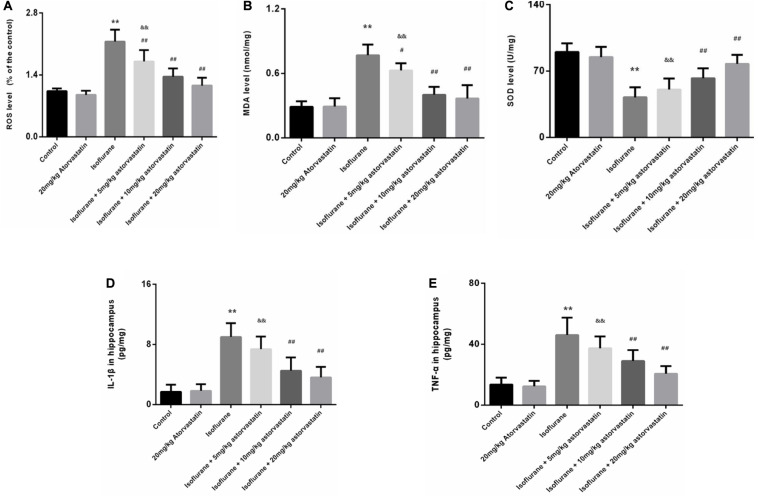
Atorvastatin inhibited oxidative stress induced by isoflurane in the aged mice. **(A)** Reactive oxygen species (ROS) levels in the hippocampus. **(B)** Malondialdehyde (MDA) levels in the hippocampus. **(C)** Superoxide dismutase (SOD) activity in the hippocampus. **(D)** The level of IL-1β in the hippocampus. **(E)** The level of TNF-α in the hippocampus. Values are represented as mean ± SD, (*n* = 8 per group). *P < 0.05 and **P < 0.01: Control Group versus Isoflurane Group, ^#^*P* < 0.05 and ^##^*P* < 0.01: Atorvastatin treatment (5 mg/kg, 10 mg/kg, and 20 mg/kg) + Isoflurane Group versus Isoflurane Group, ^&^*P* < 0.05 and ^&&^*P* < 0.01: Isoflurane + Atorvastatin with 5 mg/kg and 10 mg/kg Groups versus Isoflurane + 20 mg/kg Atorvastatin Group.

Reactive oxygen species and MDA are considered reliable indicators of oxidative stress. Superoxide dismutase (SOD) mainly eliminates superoxide anion free radicals, which are harmful to the body and play an antioxidant role. Isoflurane exposure for 6 h induced a significant increase in ROS (*F* = 46.487, *P* < 0.01, Figure 3A) and MDA (*F* = 42.448, *P* < 0.01, Figure 3B) levels in the hippocampal tissues, followed by a decrease in SOD (*F* = 27.520, *P* < 0.01, Figure 3C). Interestingly, we observed suppression of ROS and MDA levels, with increased SOD levels upon atorvastatin pretreatment (*P* < 0.01, Figures 3A–C). This suppression of ROS and MDA could possibly be due to the antioxidant effects of atorvastatin. Mice administered with atorvastatin alone (without isoflurane exposure) exhibited a slight decrease in ROS levels compared with control mice, with no statistical significance (*P* > 0.05). In addition, the dose of 10 and 20 mg/kg atorvastatin had more significant effects than those of 5 mg/kg (*P* < 0.05).

We also examined the levels of IL-1β and TNF-α in the hippocampus. Exposure to isoflurane caused an increase in IL-1β and TNF-α expression (*F*_*IL–*1β_ = 32.365, *P* < 0.01; *F*_*TNF–*α_ = 29.116, *P* < 0.01, Figures 3D,E). Administration of atorvastatin before anesthetic exposure resulted in a considerable decrease in the expression of the two cytokines compared with that in the isoflurane-alone group (*P* < 0.01, Figures 3D,E). Furthermore, atorvastatin at 10 mg/kg and 20 mg/kg was more effective than at 5 mg/kg (*P* < 0.05, Figures 3D,E). Interestingly, IL-1β and TNF-α expression levels in atorvastatin-alone treated mice that were not exposed to isoflurane were not different compared with that in normal control mice (*P* > 0.05), reflecting the absence of stress stimuli.

### Atorvastatin Inhibited the Activation of the p38MAPK/ATF2 Signaling Pathway Induced by Isoflurane

**FIGURE 4:**
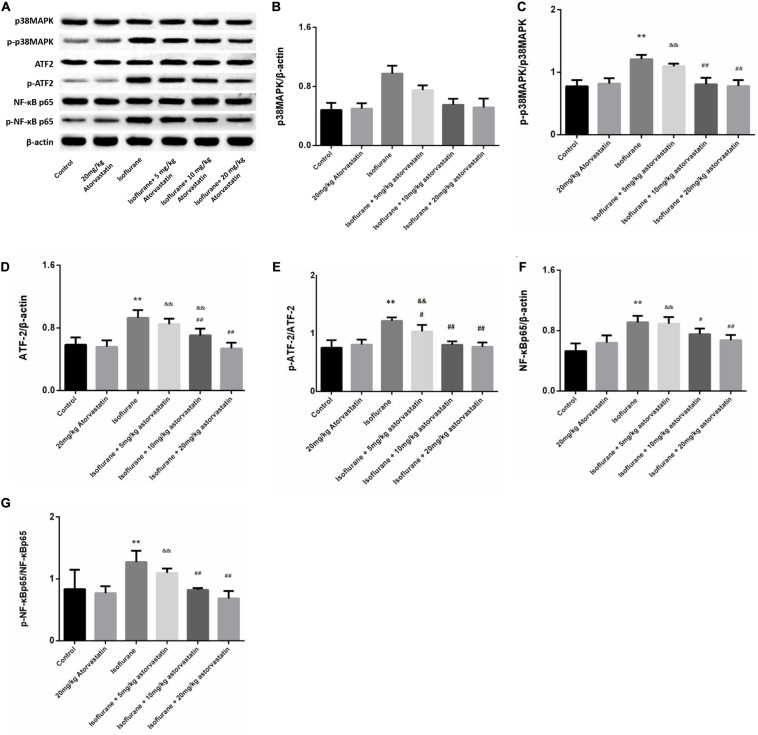
Atorvastatin modulated the p38MAPK/ATF2 signaling pathway. **(A)** Representative western blotting of the p38MAPK/ATF2-NF-κB pathway proteins, including p38MAPK, p- p38MAPK, ATF2, p-ATF2, NF-κB, p- NF-κB, and IκBα. **(B–G)** Quantitative analysis of these protein levels in the hippocampus among different groups. Isoflurane could activate p38MAPK, ATF2, and NF-κB proteins. Atorvastatin pretreatment significantly reduced expression of p-p38MAPK, p-ATF2 and p- NF-κB. Values are represented as mean ± SD, (*n* = 8 per group). *P < 0.05 and **P < 0.01: Control Group versus Isoflurane Group, ^#^*P* < 0.05 and ^##^*P* < 0.01: Atorvastatin treatment (5 mg/kg, 10 mg/kg, and 20 mg/kg) + Isoflurane Group versus Isoflurane Group, ^&^*P* < 0.05 and ^&&^*P* < 0.01: Isoflurane + Atorvastatin with 5 mg/kg and 10 mg/kg Groups versus Isoflurane + 20 mg/kg Atorvastatin Group.

In the present study, we assessed whether the reduction in neuronal apoptosis induced by atorvastatin involves the p38MAPK/ATF2-NF-κB signaling pathway. Western blotting analysis revealed enhanced expression of phosphorylated forms of p38MAPK (*F* = 39.825, *P* < 0.01, Figures 4A,C), ATF2 (*F* = 33.883, *P* < 0.01, Figures 4A,E), and NF-κB (*F* = 14.416, *P* < 0.01, Figures 4A,G) following isoflurane exposure, suggesting activation of these pathways. Levels of total p38MAPK (*P* > 0.05, Figures 4A,B), ATF2 (*P* > 0.05, Figures 4A,D), and NF-κB (*P* > 0.05, Figures 4A,F) did not changed among these groups. Atorvastatin pretreatment suppressed the activation of the p38MAPK/ATF2-NF-κB signaling pathway (*P* < 0.05) in a dose-dependent manner. These observations suggest that atorvastatin modulates the crucial pathways involved in cell survival and apoptosis.

### Effects of Isoflurane and SB203580 on BV-2 Cell Viability and LDH Release

**FIGURE 5:**
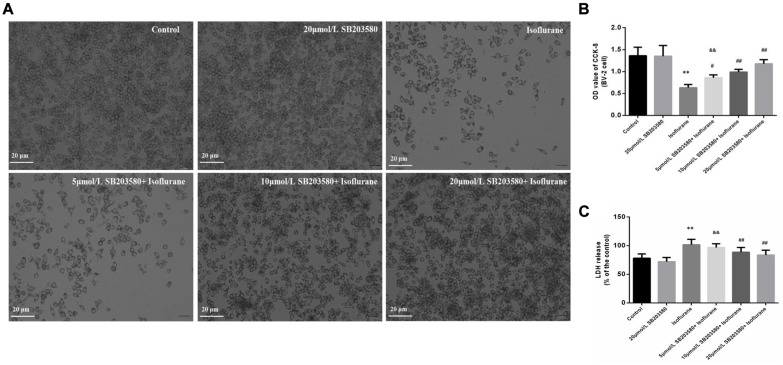
Effects of isoflurane and SB203580 on the viability of BV-2 cells and LDH release. **(A)** The morphology of BV-2 cells under an inverted/phase-contrast microscope. **(B)** The OD values of CCK-8 in BV-2 cells. **(C)** The activity of LDH release in BV-2 cells. SB203580 could prevent isoflurane induced cytotoxicity. Values are represented as mean ± SD, (*n* = 8 per group). *P < 0.05 and **P < 0.01: Control Group versus Isoflurane Group, ^#^*P* < 0.05 and ^##^*P* < 0.01: SB203580 treatment (5,10, and 20 μmol/mL) + Isoflurane groups versus Isoflurane groups, ^&^*P* < 0.05 and ^&&^*P* < 0.01: Isoflurane + SB203580 with 5 μmol/mL and 10 μmol/mL Groups versus Isoflurane + 20 μmol/mL SB203580 Group.

In this part, BV-2 cells were used to further discuss the mechanism. Cell viability was determined by the CCK-8 method and LDH release. The morphology of BV-2 cells was photographed under an inverted/phase-contrast microscope (Figure 5A). Results showed that treatment with 1.5% isoflurane for 6 h significantly reduced the OD value of CCK-8 and increased LDH release, which reflected the cell viability (*F*_*CCK–*8_ = 33.613, *P* < 0.01; *F*_*LDH*_ = 15.647, *P* < 0.01; Figures 5B,C). However, SB203580, the inhibitor of p38MAPK significantly attenuated isoflurane-induced cell apoptosis, as evaluated by CCK8 and LDH release at the three doses of 5, 10, and 20 μmol/ml (*P* < 0.05, Figures 5A–C), However, there were no significant differences in the effects of the 10 and 20 μmol/mL doses group (*P* > 0.05, Figures 5A–C), rather than the dose of 5 μmol/ml.

### SB203580 Ameliorated Isoflurane-Induced Oxidative Stress and Neuroinflammation in BV-2 Cells

**FIGURE 6:**
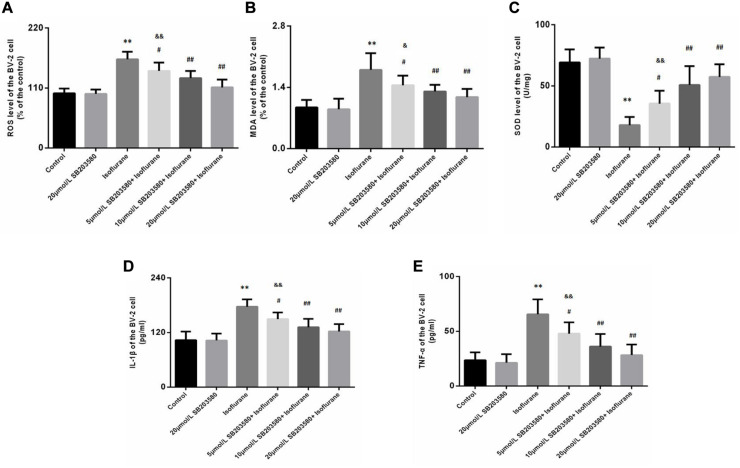
SB203580 inhibited oxidative stress induced by isoflurane in BV-2 cells. **(A)** Reactive oxygen species (ROS) levels in BV-2 cells. **(B)** Malondialdehyde (MDA) levels in BV-2 cells. **(C)** Superoxide dismutase (SOD) activity in BV-2 cells. SB203580 could inhibit isoflurane induced oxidative stress and inflammation in BV-2 cells. Levels of IL-1β **(D)** and TNF-α **(E)** in BV-2 cells. Values are represented as mean ± SD, (*n* = 8 per group). *P < 0.05 and **P < 0.01: Control Group versus Isoflurane Group, ^#^*P* < 0.05 and ^##^*P* < 0.01: SB203580 treatment (5,10, and 20 μmol/mL) + Isoflurane groups versus Isoflurane groups, ^&^*P* < 0.05 and ^&&^*P* < 0.01: Isoflurane + SB203580 with 5 and 10 μmol/mL Groups versus Isoflurane + 20 μmol/mL SB203580 Group.

Isoflurane exposure for 6 h induced a significant increase in ROS (*F* = 32.209, *P* < 0.01, Figure 6A) and MDA (*F* = 16.021, *P* < 0.01, Figure 6B) levels in BV-2 cells, followed by a decrease in SOD levels (*F* = 29.670, *P* < 0.01, Figure 6C). Interestingly, we observed suppression of ROS and MDA levels with increased SOD levels upon SB203580 pretreatment (*P* < 0.01, Figures 6A–C). In BV-2 cells treated with SB203580 alone (without isoflurane exposure), slight decreases in ROS levels were observed compared with control mice, with no statistical significance (*P* > 0.05). In addition, the doses of 10 and 20 μmol/mL SB203580 have more significant effects than the dose of 5 μmol/ml group (*P* < 0.05).

We also discussed levels of IL-1β and TNF-α in BV-2 cells. Exposure to isoflurane caused an increase in IL-1β and TNF-α expression (*F*_*IL–*1β_ = 23.753, *P* < 0.01; *F*_*TNF–*α_ = 21.771, *P* < 0.01, Figures 6D,E). Administration of SB203580 resulted in a considerable decrease in the expression of the two cytokines, compared with the isoflurane-alone group (*P* < 0.01, Figures 6D,E). Furthermore, SB203580 at 10 and 20 μmol/mL was more effective at a dose of 5 μmol/mL (*P* < 0.05, Figures 6D,E). Interestingly, in SB203580-alone treated cells that were not exposed to isoflurane, IL-1β and TNF-α expression levels were not altered compared with those in the control group (*P* > 0.05, Figures 6D,E).

### SB203580 Suppressed the Activation of the p38MAPK/ATF2 Signaling Pathway Induced by Isoflurane in BV-2 Cells

**FIGURE 7:**
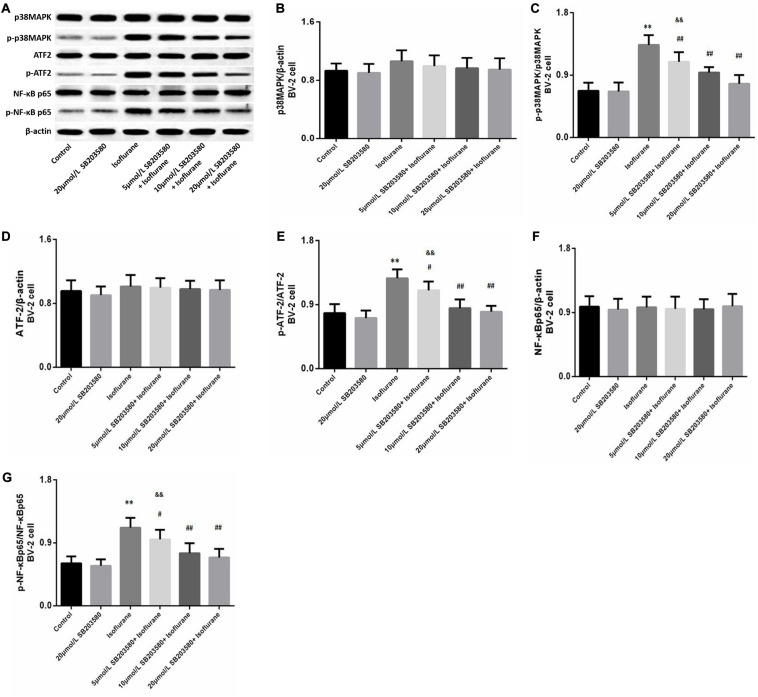
SB203580 modulated p38MAPK/ATF2 signaling pathway. **(A)** Representative western blotting of the p38MAPK/ATF2-NF-κB pathway proteins, including p38MAPK, p- p38MAPK, ATF2, p-ATF2, NF-κB and p-NF-κB. **(B–G)** Quantitative analysis of the levels of these proteins in BV-2 cells among different groups. Isoflurane could activate p38MAPK, ATF2, and NF-κB proteins of BV-2 cell. SB203580 significantly reduced expression of p-p38MAPK, p-ATF2 and p-NF-κB. Values are represented as mean ± SD, (*n* = 8 per group). *P < 0.05 and **P < 0.01: Control Group versus Isoflurane Group, ^#^*P* < 0.05 and ^##^*P* < 0.01: SB203580 treatment (5,10, and 20 μmol/mL) + Isoflurane groups versus Isoflurane groups, ^&^*P* < 0.05 and ^&&^*P* < 0.01: Isoflurane + SB203580 with 5 and 10 μmol/mL Groups versus Isoflurane + 20 μmol/mL SB203580 Group.

Western blotting analysis of BV-2 cells revealed enhanced expression of phosphorylated forms of p38MAPK (*F* = 36.939, *P* < 0.01, Figures 7A,C), ATF2 (*F* = 28.468, *P* < 0.01, Figures 7A,E), and NF-κB (*F* = 23.483, *P* < 0.01, Figures 7A,G) following isoflurane exposure, suggesting activation of these pathways. Levels of total p38MAPK (*F* = 1.326, *P* > 0.05, Figures 7A,B), ATF2 (*F* = 0.762, *P* > 0.05, Figures 7A,D) and NF-κB (*F* = 0.9352, *P* > 0.05, Figures 7A,G) were not significantly enhanced. However, SB203580 treatment inhibited the activation of the p38MAPK/ATF2-NF-κB signaling pathway (*P* < 0.05) in a dose-dependent manner. These observations illustrated that the p38MAPK/ATF2-NF-κB pathway was involved in cell survival and inflammation under isoflurane exposure.

## Discussion

Isoflurane is a common anesthetic in adult and aged patients during surgery. Nevertheless, it has been reported to induce cognitive dysfunction and neurodegenerative changes in aged brains (Wang et al., 2018; Fu et al., 2020; Wen et al., 2020). Previous studies have reported that isoflurane-induced neurodegeneration is associated with oxidative stress and neuroinflammation (Zhang and Zhang, 2018; Lee et al., 2019; Li et al., 2019). The potential mechanisms remain clear. The p38MAPK/ATF2 signaling is an important regulatory factor in cellular life activities, including inflammatory response, cell development, differentiation, as well as apoptosis (Santa-Cecília et al., 2016). Thus, the present study was performed to discuss the role of the p38MAPK/ATF2 pathway on isoflurane-induced neurotoxicity.

*In vivo* study, we found isoflurane-induced cognitive impairment in aged mice. Neuronal apoptosis was also observed in the CA1 region of the hippocampus after 6 h of isoflurane exposure, which was consistent with previous studies (Wu et al., 2019; Qiu et al., 2020). Besides, isoflurane could upregulate Bad and Bax proteins and reduce Bcl-2 level in the hippocampus. Bax translocates from the cytosol to mitochondria, promotes the release of cytochrome c, and activates the caspase cascade, while Bcl-2 blocked Bax translocation (Choi, 2020; Li W. et al., 2020; Luo et al., 2020). Caspase-3, as a primary executioner caspase enzyme, can contribute to cellular apoptosis (Sarif et al., 2020). The activation of caspase-9 and caspase-3 was observed in mice exposed to isoflurane. Thus, treatment with 1.5% isoflurane for 6 h induced neurotoxicity as previously reported (Shen et al., 2020). Although some studies showed the neuroprotective effects of isoflurane (Han et al., 2020; Tian et al., 2020), it may be due to the duration of isoflurane exposure. The results implied that prolonged isoflurane anesthesia could damage the cognition of aged patients in clinical.

Accumulating evidence indicates that oxidative stress and inflammation may contribute to anesthetic-induced neurotoxicity in the aged brain (Wu et al., 2015; Xu et al., 2020). Excessive oxidative stress and inflammation could promote the inflammatory factors release, which would damage the integrity of blood brain barrier and activate microglia, further lead to inflammatory cascades and cellular apoptosis (Otani and Shichita, 2020; Scuderi et al., 2020). In our study, 1.5% isoflurane could promote oxidative stress and inflammation in the aged brain, with an increase in ROS, MDA, IL-1β, and TNF-α as well as a decrease in SOD. These results were consistent with those of other studies (Wu et al., 2012, 2015; Wang et al., 2018), which suggested that oxidative stress was a contributing factor for isoflurane-induced neuronal death.

Atorvastatin, a 3-hydroxy-3-methylglutaryl coenzyme A reductase inhibitor, is lipophilic and easily crosses the blood-brain barrier (Sparks et al., 2002; Lübtow et al., 2020). In recent years, except for lipid-lowering effects, the anti-inflammatory and anti-oxidative stress as well as anti-apoptotic roles of atorvastatin have gained increasing attention (Li et al., 2014; Xu et al., 2017). In the present work, we found that both 10 mg/kg and 20 mg/kg atorvastatin could attenuate cognitive dysfunction and apoptosis induced by isoflurane. Furthermore, atorvastatin also ameliorated oxidative stress and neuroinflammation in a dose-dependent manner, which proved the neuroprotective effect of atorvastatin on the isoflurane-induced neurobehavior injury. These results are consistent with previous studies on other models of cognitive impairment, such as ischemia/reperfusion, Alzheimer’s disease models, etc. (Zhang et al., 2014; Shao et al., 2017; Wang et al., 2019). Thus, atorvastatin might be an effective drugs to prevent postoperative cognitive impairment.

Further, we discussed the related mechanisms for the inflammation regulation. The p38MAPK/ATF2 pathway is an important signaling pathway for inflammation activation and regulation, which can regulate the activation of NF-κB and the maturation and release of inflammatory factors (Awasthi et al., 2020). Liao et al. reported that the p38MAPK pathway is involved in isoflurane-induced hippocampal neuroapoptosis in neonatal rats (Liao et al., 2014). Another study showed the neurotoxicity of p38MAPK in APP/PS1 mice (Gong et al., 2019). In our study, we observed that isoflurane enhanced the phosphorylation levels of p38MAPK, ATF2, and NF-κB, with a decrease in IκBα activation in the hippocampus. Levels of total p38MAPK, ATF2, and NF-κB were also slightly increased. The results implied that p38MAPK/ATF2 pathway may be involved isoflurane induced neuroinflammation. Atorvastatin treatment significantly inhibit isoflurane induced activation of p38MAPK, ATF2 and NF-κB, thereby inhibiting the signaling cascades. Besides, in another study, atorvastatin could prevent memory dysfunction caused by amyloid-β peptide oligomer through p38MAPK pathway (Zhang et al., 2014).

p38MAPK is a member of the MAPKs, which are a family of serine-threonine protein kinases. The p38MAPK signaling pathway is mainly involved in the regulation of intracellular oxidative stress, inflammation, cell proliferation, and apoptosis (Maik-Rachline et al., 2020). Various physical and chemical stimuli, such as ROS, could activate classic MAP3K MKK3/6 and further phosphorylate p38MAPK. ATF2 is a critical downstream signaling molecule. Activated p38MAPK could phosphorylate the Thr69 sites of ATF2 (Tindberg et al., 2000). The activated p38MAPK/ATF2 pathway modulates the expression and activation of NF-κB and inflammatory cytokines, such as IL-1β and TNF-α (Hisatsune et al., 2008). In addition, activated p38MAPK/ATF2 is involved in apoptosis (Yan et al., 2013). Although we found that atorvastatin could inhibit the activation of p38MAPK/ATF2 pathway induced by isoflurane, the role of p38MAPK/ATF2 pathway in isoflurane-induced neurotoxicity remains unclear.

Consequently, to determine whether p38MAPK/ATF2 was associated with isoflurane-induced neuroinflammation and oxidative stress, BV-2 cells were used *in vitro* study. Microglial functions are susceptible to damage in the aging brain, which is followed by inflammatory activation leading to promotion of neurodegeneration and cognitive dysfunction (Brites and Fernandes, 2015; Vincenti et al., 2015; Fougère et al., 2017). Activated microglia can modulate cytokine release, cell apoptosis, and neuronal plasticity (Vincenti et al., 2015; Santarsiero et al., 2020). In addition, previous studies have used BV-2 cells to discuss the isoflurane-induced neurotoxicity (Tanaka et al., 2013; Yang et al., 2016; Wang et al., 2018). Thus, BV-2 cells were used in this study.

SB203580, a p38MAPK inhibitor, has been widely used in the study of the p38MAPK pathway (Liao et al., 2014; Li T. et al., 2020; Liu et al., 2020; Yang C. et al., 2020). In our study, results showed that isoflurane could inhibit cell viability detected by the CCK8 and LDH activity kits, while SB203580 reversed the cytotoxicity of isoflurane in a dose-dependent manner with attenuating the phosphorylation of p38MAPK, ATF2, and NF-κB and levels of two cytokines. The effects of SB203580 treatment with 20 μmol/L were the most obvious. The results showed that the ROS-p38MAPK/ATF2 pathway was associated with isoflurane induced neuroinflammation, apoptosis, and memory damage. In addition, SB203580 decreased the levels of ROS and MDA and increased the expression of SOD, which implied that there was a positive interaction between the ROS system and the p38MAPK/ATF2 pathway. Both the ROS system and p38MAPK/ATF2 pathway are involved in the regulation of isoflurane-induced cognitive impairment in aged mice. Atorvastatin inhibited ROS system, the p38MAPK/ATF2 pathway, and further played an anti-inflammatory and neuroprotective role in the isoflurane-induced neurotoxicity.

There are also some limitations to our study. First, in this study, we mainly discussed the changes of related indicators in the hippocampus and hippocampal related memory and not in other brain regions, including prefrontal cortex, hypothalamus, etc. Second, this study mainly discussed short-term cognitive functions after isoflurane, rather than long-term changes. The behavioral tests implied that cognitive impairment persisted 6–7 days after 1.5% isoflurane exposure. Third, *in vivo* study, we discussed the dose-dependent effects of atorvastatin on cognitive impairment, without SB203580 treatment because the aged mice are hard to get currently. *In vitro* experiment, we did not discuss the neuronal changes that occurred when cells were exposed to isoflurane and atorvastatin; this will be explored in future studies.

## Conclusion

**FIGURE 8:**
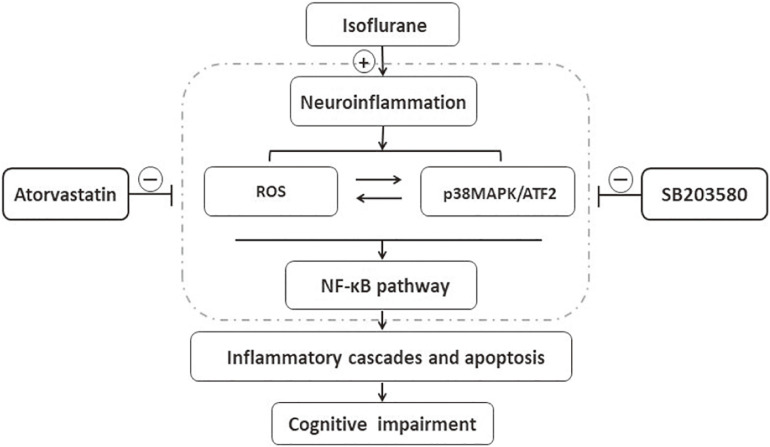
Schematic representation of the neuroprotective effects of atorvastatin following isoflurane exposure. Isoflurane induced oxidative stress and neuroinflammation, which could activate the p38MAPK/ATF2-NF-κB signaling pathway, and further led to inflammatory cascades, neuronal apoptosis, and cognitive impairment. SB203580, a selected p38MAPK inhibitor, attenuated inflammatory cascades, oxidative stress, and neuronal apoptosis, which implied that ROS-p38MAPK/ATF2 pathway was involved in the isoflurane-induced neurotoxicity. Besides, atorvastatin treatment effectively attenuated isoflurane-induced oxidative stress, inflammation cascades, and cognitive impairment, partly by inhibiting ROS-p38MAPK/ATF2 pathway.

The results of this study implied that isoflurane induced oxidative stress and neuroinflammation by activating the ROS-p38MAPK/ATF2 pathway. Moreover, it induced apoptosis and cognitive impairment in aged mice. However, SB203580 treatment could attenuate isoflurane-induced cytotoxicity. Besides, atorvastatin treatment also effectively attenuates isoflurane-induced oxidative stress and inflammation, partly by inhibiting the ROS-p38MAPK/ATF2 pathway Figure 8. The findings illustrated the neuroprotective effect of atorvastatin against isoflurane-induced neurotoxicity. In addition, p38MAPK might be a potential therapeutic target for cognitive impairment after anesthesia and surgery.

## Data Availability Statement

The raw data supporting the conclusions of this article will be made available by the authors, without undue reservation.

## Ethics Statement

The animal study was reviewed and approved by the ethical committee of the Beijing Shijitan Hospital, Capital Medical University.

## Author Contributions

TL conceived and designed this study. XX and SL optimized experiment, provided technical support, and coordinated personnel arrangement. PL and WS drafted the manuscript. Material preparation, data collection were performed by TG, JJ, YH, and YX. QSG, LG, and HQ corrected the manuscript and contributed the materials and analysis tools. All the authors read and approved the final manuscript.

## Conflict of Interest

The authors declare that the research was conducted in the absence of any commercial or financial relationships that could be construed as a potential conflict of interest.

## Abbreviations

ANOVA: analysis of variance
ATF2: activating transcription factor 2
CCK-8: cell counting kit-8
DMEM: Dulbecco’s modified Eagle’s medium
DMSO: dimethylsulfoxide
ELISA: enzyme-linked immunosorbent assays
IκB: inhibitor of NF-κB
IL-1β: interleukin-1 beta
JNK: c-junN-terminal kinase
LDH: lactate dehydrogenase
MAPK: mitogen-activated protein kinase
MDA: malondialdehyde
MWM: Morris water maze
NF-κB: nuclear factor kappa-B
OF: open field
RIPA: radio immunoprecipitation assay
ROS: reactive oxygen species
SD: standard deviation
SOD: superoxide dismutase
TNF-α: tumor necrosis factor-α
TUNEL: terminal deoxynucleotidyl transferase-mediated dUTP nick-end labeling.
